# Direct and indirect effects of pesticides on a benthic grazer during its life cycle

**DOI:** 10.1186/s12302-018-0165-x

**Published:** 2018-09-17

**Authors:** Marcus Rybicki, Dirk Jungmann

**Affiliations:** Institute of Hydrobiology, Technische Universität Dresden, Zellescher Weg 40, 01217 Dresden, Germany

**Keywords:** Terbutryn, Lambda-cyhalothrin, Mayfly, Aufwuchs, Artificial indoor streams, Mesocosm, Microcosm, Trophic interactions, Time-shifted exposure

## Abstract

**Background:**

Macroinvertebrates in aquatic ecosystems are repeatedly exposed to pesticides during their life cycle. Effects of consecutive exposure during different life stages and possible synergistic effects are not addressed in the standardized hazard assessment. The present study investigated two environmentally relevant exposure scenarios in batch (microcosm) and artificial indoor stream (mesocosm) experiments using the larvae of the mayfly *Rhithrogena semicolorata* (grazer) and natural aufwuchs. Grazers were analysed regarding growth, physiological condition, and drift behaviour, while the aufwuchs was analysed in terms of biomass using the particulate organic carbon as well as the chlorophyll a content. The aim was to reveal direct and indirect effects of an herbicide exposure during autumn on juvenile grazers and an insecticide exposure during spring on semi-juvenile grazers.

**Results:**

Direct and indirect effects were found in both exposure scenarios at environmentally relevant concentrations. In the herbicide exposure scenario with terbutryn, clear direct effects on the aufwuchs community with a LOEC of 0.38 µg L^−1^ were found. Effect levels of grazers due to indirect effects were equal, with the overnight drift being the most sensitive grazer endpoint. In the insecticide exposure scenario, clear lethal and sub lethal effects of lambda-cyhalothrin were evident. Derived LC_50_ values for the artificial indoor stream and batch experiment were 2.42 µg g^−1^ OC (69 days) and 1.2 µg g^−1^ OC (28 days), respectively. Sub lethal effects in terms of increased drift as well-reduced growth and triglyceride levels were found at concentrations of 1.4 and 0.09 µg g^−1^ OC (LOECs). These results were confirmed by the batch experiment, which revealed effect values in the similar range. Finally, a clear indirect effect of the insecticide on the aufwuchs was evident in the batch experiment with an LOEC at 0.9 µg g^−1^ OC.

**Conclusion:**

Toxicity Exposure Ratios calculated with the derived effect values indicate a risk for the investigated grazer by both pesticides. Moreover, observed indirect effects during the herbicide exposure seem to be able to affect the grazers during a second exposure with an insecticide, due to reduced physiological conditions. We suggest further research with time-shifted exposure scenarios to gain a better understanding of the complex interactions of pesticides with the life cycle and the food webs of macroinvertebrates.

## Background

Pesticides are widely used within the industrialized agriculture as shown by the great amounts that are worldwide applied (ca. 395,944.4 t a^−1^ in Europe 2014—Eurostats, Pesticide sales Reg. 1185/2009, [54]). The majority of the applied pesticides in Europe are fungicides and bactericides (44%) followed by herbicides (33%). Insecticides and acaricides still share 5% of the total amount, which corresponds to 20,706.3 t a^−1^ in entire Europe in 2014. In spite of intensified efforts in terms of regulation and technological advances for environmental risk mitigation, recent studies revealed that pesticides still massively contribute to the toxicity to non-target organisms within surface waters in Europe [37, 53]. Regular monitoring programs of surface waters revealed thereby that pesticides do not occur equally during the year, but appear to specific seasons, which, of course, correlates with respective agricultural activities. In late summer to autumn, primarily herbicides are used in terms of pre harvest application (siccation), post-harvest preparation, pre emergence treatment, as well as for weed treatment within the crops. In spring and early summer, predominantly insecticides are used to control emerging insect pests. Therefore, non-target organisms within the aquatic environment are regularly exposed to different pesticides during respective developmental stages of their life cycle. Consequently, the seasonal exposure pattern may cause harmful effects either directly or indirectly, which pose a specific risk for insects with uni- or paravoltine lifecycles in the aquatic environment.

The current study aimed on effects of the described exposure scenario on the important ecosystem function of benthic grazing performed among others by larvae of mayflies. Benthic grazing is extraordinarily important in small- and medium-sized streams to control the biomass of aufwuchs. Especially, in spring before foliation of deciduous trees [28, 30, 58], the autotrophic components of the aufwuchs community, the microphytobenthos or periphyton, increases fast in terms of biomasses, due to the increasing light availability and the higher water temperatures in the streams. This process is fostered by eutrophication and can result in increased external biotic colmation [30, 50], which hampers the infiltration of stream water into the hyporheic interstitial with negative consequences for the entire aquatic ecosystem [11, 30, 70]. Benthic grazers are capable to reduce and even control the growth of aufwuchs during this time if the required biomasses are achieved [4, 18, 22, 29, 31]. Important grazers are larvae of the mayfly *Rhithrogena semicolorata* (Curtis, 1834; Ephemeroptera:Heptageniidae). They achieve high biomasses in streams of central Europe and contribute, therefore, essentially to this ecosystem function. *R.* *semicolorata* performs a univoltine winter cycle, i.e., after egg deposition in May–June, the larvae develop within the egg over the summer and hatches in late summer. Thereafter, they grow during autumn and winter [16] and utilize the aufwuchs biomass peak in spring by intense grazing to grow substantially and finally achieve adulthood. Hence, considering the above-mentioned exposure situation and their development reveals a first exposure to herbicides during the sensitive young larval stage in autumn and a second exposure to insecticides during spring, where grazers are supposed to perform the grazing effectively.

In the present study, this exposure scenario, which concerns different developmental stages of the mayfly life cycle, was simulated in a tiered approach. Simple microcosm batch and higher tier artificial indoor stream (AIS) experiments were performed to investigate the effects of solely herbicide or insecticide exposure to larvae of *R.* *semicolorata* at the respective time of their life cycle. The experiments presented in this study represent the first step of a set of experiments with the overall aim of a combined time-shifted exposure, as it occurs in the environment. The aim of the present experiments was, therefore, to first reveal direct and indirect effects of the single herbicide and insecticide exposure on the respective developmental stages of the grazers and identify potential synergistic effects in case of combined time-shifted exposure as it occurs in the environment.

The first experiment performed in autumn/winter investigated the effects of environmentally relevant concentrations of the herbicide terbutryn. In previous studies, it was already shown that terbutryn affects the quantity and quality of aufwuchs [12, 52]. Moreover, negative impacts affecting the physiological fitness of the grazer *R.* *semicolorata* were found, due to indirect effects [52]. The present study increased the complexity of the experimental design by including further environmental stressors like stream velocity and further endpoints like grazer drift into the effect analysis. The hypothesis was that environmentally relevant concentrations of terbutryn are able to affect the development of early life stages of *R.* *semicolorata* negatively, due to food shortage and reduced food quality.

The second set of experiments, consisting of a batch and an AIS experiment, aimed on the older life stages of *R. semicolorata* and the potential effects of environmentally relevant insecticide concentrations. As insecticide, the pyrethroid lambda-cyhalothrin (LCH) was chosen, which is often used, e.g., in the culture of rape, corn, and potatoes [59]. LCH is very toxic to invertebrates and fishes [14, 20, 55]. Due to its high lipophilicity, it dissipates rapidly from the water phase to organic matter, e.g., aufwuchs or sediments [35, 39] and is, therefore, expected to affect especially grazing organisms like *R.* *semicolorata* via oral uptake (food) and the contact to contaminated aufwuchs. Apart from increased mortality [51, 61], the experiments focused specifically on sublethal effects to reveal negative impacts of environmentally relevant insecticide concentrations on the development of *R.* *semicolorata*. We hypothesized that environmentally relevant concentrations of LCH causes significant behavioural changes, which results in increased drift and reduced feeding [25, 38, 43].

## Methods

### Study organisms and analysis

We used the grazer-aufwuchs-interaction as a model, consisting of the benthic grazer *R.* *semicolorata* and benthic aufwuchs collected in the field. All organisms used were sampled at the Gauernitzbach near Dresden (Germany; 51°06′N, 13°32′E) in December 2009 and March 2010. The Gauernitzbach is a second-order mountain stream moderately affected by agricultural runoff but intensively monitored, due to several ecological experiments of the Institute of Hydrobiology, TU Dresden [69, 71].

### Grazers

Larvae of *R.* *semicolorata* were obtained by kick sampling [66] 2–5 days before introduction into respective experiments. Caught organisms were transferred to plastic boxes filled with stream water and transported to the laboratory immediately, where they were kept in aerated glass tanks at ambient temperatures (10–15 °C) in a greenhouse until use.

For grazer sampling, as scheduled in the section Experimental design, the grazers were immediately transferred to vials (1.5 mL, Eppendorf) and frozen in liquid nitrogen or in an ultra-low freezer (− 80 °C, MDF382, SANYO, Japan). All grazers were lyophilized (24 h, ca. − 150 °C, alpha 1–2, Christ) and weighted with an ultra-sensitive balance (M3P, Sartorius, Germany). Grazer triglyceride content at specific dates were determined utilizing an enzymatic essay (Sigma-Aldrich, USA) and a random subsample (*n* = 6 per treatment) of grazers. Triglycerides from lyophilisated and weighed larvae were extracted according to Winkelmann and Koop [68], but with a reduced extraction volume of 0.5 mL extraction solvent (hexanol:isopropanol mixture, 3:2, v:v). Subsamples of the extract (10, 25, or 75 µL) were transferred into 96-well microplates (96/F-PP, Eppendorf; 3–4 replicates per sample) and afterwards placed in a laboratory hood for 12 h for evaporation of the extraction solvent. For triglyceride determination, 200 µL of work reagent (triglyceride reagent [T2449] + free glycerol reagent [F6428], 1:4, v:v, both Sigma-Aldrich) were added to respective wells. Microplates were incubated at 30 °C and measured after 120 min with a plate reader (2010, Anthos Mikrosysteme, Germany) at 540 nm. A glycerol standard (G7793, Sigma-Aldrich) was additionally analysed on each plate after 10 min, due to faster reaction of the enzymatic assay with the standard. This standard was also used for the calibration of the method (0.0028–0.14 µMol Well^−1^ triglyceride equivalents).

As further sub lethal parameter in the AIS experiments, the overnight drift was determined every morning at 9 A.M. by counting of grazers caught in the protective gratings (drift nets) of the AIS. Caught grazers were repositioned on the tiles of the AIS after counting or removed and frozen if heavily injured or dead. Grazers sampled from the drift nets (irregular sampling) were considered at the next regular sampling dates to keep the total grazer density within each AIS constant. The drift data were analysed as relative cumulative drift over the exposure period in relation to the respective abundance of grazers in the AIS at each day. Finally, the total mortality was calculated at the end of the respective experiment by addition of lost and injured grazers compared to the initial number.

### Aufwuchs

Aufwuchs was established on unglazed ceramic tiles (4 × 6 cm) according to Bohle [8] as described in Rybicki et al. [52]. After this procedure, the tiles were covered with a slight equal layer of aufwuchs and ready for introduction into the AIS for further growth of aufwuchs.

The aufwuchs biomass was quantified by the analysis of particulate organic carbon (POC) at the respective sampling dates. In the terbutryn experiment, additionally, the chlorophyll a content was analysed. Aufwuchs was sampled by scraping it with a merchantable tooth brush and 50 mL of tap water from the tiles into a bowl. A subsample of 1–10 mL of the resulting aufwuchs suspension, depending on the aufwuchs biomass, was filtered over pre-ashed glass fibre filters (500 °C > 45 min, MGF, 25 mm diameter, Sartorius) for the analysis of POC. Standard glass fibre filters of the same type were used for chlorophyll a analysis. Filtration was carried out using glass filtration equipment, a vacuum pump, and under pressure of − 0.2 bar. Filters for POC analysis were dried at 70 °C for 24 h and stored in a desiccator until analysis in a carbon analyzer (C-200, Leco, USA). Filters for chlorophyll a analysis were placed in vials (1.5 mL, Eppendorf, Germany) and quickly frozen in a freezer. To prevent degradation of the light sensitive chlorophyll pigments, filters were stored light protected at − 22 °C in a freezer for at least 24 h until analysis. Furthermore, all working steps of the chlorophyll a analysis were performed in the dark. Chlorophyll a extraction occurred after lyophilisation of filters (ca. 12 h at ca. − 150 °C, alpha 1–2, Christ, Germany). Filters were shredded with a scissor and homogenised in 25 mL of Ethanol (90%, buffered with 1 g L^−1^ MgCO_3_, Merck) in an ultra-dispenser (T18 basic, ULTRA-TURRAX, IKA, Germany) at 14,000 rpm for 2 min. After 24 h of extraction in brown glass bottles, the suspensions were filtered through a glass frit (pore size 1.6 µm) using under pressure (ca. − 0.8 bar). Chlorophyll a concentration of the filtered samples (25 ml) was determined by quantification of fluorescence at 667 nm after excitation at 434 nm with a fluorescence spectroscope (LS 50B, Perkin-Elmer, USA). A correction against degraded chlorophyll a (pheophytin) was made by a second quantification 5 min after acidification of filtered samples (25 mL) with 100 µL of HCl (2 M, Merck). Chlorophyll a content of aufwuchs on the tiles was calculated according to Wetzel and Likens [65].

### Experimental design

**Fig. 1:**
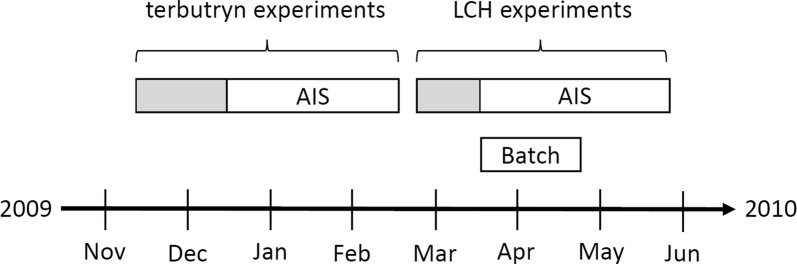
Timing of the different experiments. Grey areas of the AIS experiments indicate the pre-application period, the white areas are the exposure periods

To investigate the effects of the pesticides, generally, two kinds of experiments were performed. For terbutryn, a mesocosm experiment in Artificial Indoor Streams (AIS) was conducted. The effects of LCH were investigated by an AIS experiment and, additionally, by a batch experiment (Fig. 1).

### Artificial indoor streams

To simulate lotic conditions of running waters, we performed mesocosm experiments in five stainless steel artificial indoor streams (AIS) located in a greenhouse at the Institute of Hydrobiology at the TU Dresden [32]. Each stream was divided into two flow channels to provide basic error estimation within each stream via pseudo-replication. The experiments consisted of two periods, an initial pre-application period (d_− 22_ to d_− 1_) to establish aufwuchs biomass on unglazed ceramic tiles (4 × 3 cm) and adapt the grazers to the conditions in the AIS and the exposure period (d_0_ to d_69_), where grazers, aufwuchs, and the respective pesticide were present.

24 h before the start of the pre-application period, the AIS were filled with 500 L of modified Borgmann media ([9], LO4-S and additives E + H), prepared from particle and charcoal filtered tap water. Circulation was started immediately after filling to ensure the evaporation of chlorine residues from tap water and equilibrium of the modified Borgmann media. Whereas the water temperature was kept at the technically lowest possible temperature of the AIS of 6 °C in the terbutryn experiment to simulate winter conditions, the water temperature was risen in the LCH experiment from initial 6 °C up to the 10 °C by 1 K every 2 weeks starting at d_13_ to account for temperature increase in streams during spring. Flow velocity was set to 0.15 ms^−1^ in the middle of water column in both AIS experiments, which has been proven to be a suitable flow velocity for *R.* *semicolorata* with only moderate drift activity in preliminary experiments. Nutrients for the growth of aufwuchs were added in both experiments at least once with the preparation of the modified Borgmann media (2 mg L^−1^ Nitrate-N as NaNO_3_ [Merck, 99.5%]; 5 mg L^−1^ Silicate-Si as Na_2_SiO_3 _× 5 H_2_O [Fluka, 97%]; 0.01 or 0.02 mg L^−1^ Phosphate-P as Na_2_HPO_4_ [Merck, 99%]).

At the beginning of the pre-application period (d_− 22_), 60 aufwuchs covered tiles were randomly chosen from the growth aquaria and carefully placed into each AIS flow channel (in total 120 per AIS, see above). Grazers were added to the AIS in an abundance of one grazer per tile at d_− 8_ in the LCH experiment and d_−1_ in the terbutryn experiment, which corresponds to 446 grazers per m^2^. Only healthy and vital animals were randomly chosen and transferred to the AIS. Within the first 48 h deceased grazers were replaced (2 in the terbutryn experiment/11 in the LCH experiment). The exposure period started at d_0_ with the application of terbutryn or LCH to the streams and was finalized at d_69_. Regular sampling dates for aufwuchs and grazers (if already introduced) were d_− 15_, d_− 8_, d_0_, d_6_, d_13_, d_27_, d_41_, d_55_, and d_69_. Basic physicochemical characteristics (conductivity, temperature, pH, and oxygen) in the AIS were measured twice a week and nutrients (SRP–P, NO_3_–N, and TAN–N) at least once a week following the method described in Rybicki et al. [52].

### Batch experiment

In parallel to the LCH AIS experiment, a microcosm batch experiment with four LCH concentrations and a control was performed under laboratory conditions in 2 L glass beakers. Aufwuchs covered tiles were taken from the AIS during pre-application period (d_− 8_). Grazers were from the same sampling as for the AIS experiment. In the first phase of the experiment, 8 aufwuchs covered tiles per treatment were placed in glass tanks (35.5 × 23 × 25.5 cm) with 3 L of modified Borgmann media. The respective LCH stock solutions were added into the tanks, and equal distribution of the substance was reached by aeration with Pasteur pipette (glass) and an air pump. The tiles remained in the tanks for 24 h to allow LCH to bind to the aufwuchs. Afterwards, the tiles were slightly dipped in unpolluted modified Borgmann media for washing and always two tiles were transferred into a glass beaker filled with 1 L of unpolluted modified Borgmann media. The beakers were placed in a water bath with a cooling unit (Compatible Control, Huber, Germany) and were aerated with Pasteur pipettes and an air pump. The exposure phase started with the introduction of 4 grazers into each beaker and was terminated after 28 days. Basic physicochemical parameters were measured weekly as in the AIS experiment. Grazer and aufwuchs analysis was performed at the end of the experiment.

### The herbicide terbutryn

Terbutryn [2-(tert-butylamino)-4-(ethylamino)-6-(methylthio)-1,3,5-Triazin; CAS: 886-50-0] (Dr. Ehrenstorfer GmbH, Germany, 98% purity), a synthetic herbicide of the group of symmetric triazines, was used as herbicide in the AIS experiment. It was used as an herbicide against floating aquatic plants [41, 42] and for weed protection in agriculture [48]. Although terbutryn lost approval for use as agricultural herbicide in Germany in 2003 (European Commission Regulation 2076/2002), it has recently been found with concentrations up to 5.6 µL^−1^ in German surface waters [12, 48]; ARGE [5]. Furthermore, it is still used as a biocide in Germany according to EU directive 98/8/EG, e.g., in antifouling paints and coatings and is, therefore, still relevant. Terbutryn inhibits the photosystem II by binding on the plastoquinone-binding domain in the thylakoid membrane and thus prevents the electron transport from the photosystem to plastoquinone [60]. Terbutryn has a log K_OW_ of 3.5 and is stable against hydrolysis at a pH range of 5–9. The water solubility is 25 mg L^−1^ (20 °C).

In the AIS experiment, terbutryn was applied once at d_0_ dissolved in 25 mL ethanol (purity 100%, BDH Prolabo—VWR International, Germany) at concentrations of 0.006, 0.06, 0.6, and 6 µg L^−1^, which are the treatments of this experiment. The control stream was treated with 25 mL of ethanol only. Terbutryn was analysed in the water phase at d_0_ (1 h after application) as well as on d_28_ and d_68_ of the AIS experiment. For this purpose, 1 L of water was sampled from each AIS, filtered through a glass fibre filter (glass microfibre 696, VWR International), and extracted using solid-phase extraction (SPE) as described in Rybicki et al. [52]. SPE cartridges were stored in the refrigerator at 4 °C until analysis.

Terbutryn was quantified using gas chromatography and MS detection (GC 7890A & MS 5975C, Agilent) with an Agilent HP5-MS capillary column (30 m length; 0.25 mm inner diameter and 0.25 µm film thickness). Helium was used as the carrier gas (5.0, AIR LIQUIDE) with a flow rate of 1 mL min^−1^. The injector operated in splitless mode at 280 °C (isothermal) with a split off time of 1 min and 1 µl of each purified and concentrated extract (200 µL reconstitution volume with solvent ethyl acetate) was injected. The GC oven temperature started at 70 °C for 2 min and then increased in 20 °C min-1-steps to 280 °C, which was held for 4 min resulting in a total run time of 27 min. Terbutryn had a retention time of 19.57 min. After electron impact ionization with 70 eV (ion source temperature 230 °C and quadruple temperature 150 °C), the quantification was performed via ion 226 *m/z* and the qualification via ion 185 and 241 *m/z*. The limit of detection (35 pg µL^−1^) and the limit of quantification (150 pg µL^−1^) of the instrument were calculated according to DIN 32645 [15].

### The insecticide lambda-cyhalothrin

Lambda-cyhalothrin [LCH, CAS: 91465-08-6] is a synthetic pyrethroid of the second generation and approved as insecticide in agriculture under the European Council Directive 91/414/EEC. Reported EC_50_ values for immobilisation range from 310 ngL^−1^ (*Daphnia magna*, 48 h) to 16 ngL^−1^ (EC_50_ *Gammarus pulex* neonates, 48 h). For the mayfly *Cloeon dipterum,* Schroer et al. [55] reported an LC_50_ value (96 h) of 105 ngL^−1^. LCH has a log K_OW_ of 7 and a very low water solubility of only 5 µL^−1^ (20 °C). Due to these physicochemical properties, LCH dissipate rapidly from the water phase into surfaces like sediment or organic biofilms. Reported dissipation times (DT_50_) are in a range of 5–24 h [17, 35, 39]. From literature reviews and preliminary tests, we defined LCH water concentrations for the AIS experiment of 0.01, 0.1, 1, and 10 ng L^−1^. We chose concentrations below the reported acute affect levels, as we primarily aimed on sub lethal effects of LCH on grazers.

For the present experiments, technical grade LCH (98.5%, Dr. Ehrenstorfer, Germany) was solved in ethanol (96%, MERCK) for respective stock solutions. To achieve these concentrations, LCH was applied at d_0_ as stock solutions to the respective AIS. The control stream was treated with 5 mL ethanol. Due to rapid absorption of LCH to surfaces and varying surface–volume ratios between the performed experiments, the usage of water concentrations alone was not appropriate. We estimated from the previous studies that the majority of LCH binds to the biofilm covered tiles, as other surfaces were made of stainless steel (AIS experiments) or glass (batch experiments) with insignificant amounts of unintended biofilms and detritus at time of exposure. Hence, loads of 0.003, 0.03, 0.3, and 3 ng LCH cm^−2^ tile surface were calculated for the AIS experiment. After determination of the carbon content (POC) of the aufwuchs on the tiles, additionally, LCH loads per gram carbon (µg LCH g^−1^ OC) for the different treatments were calculated for the time of exposure to enable comparison of determined effect levels with the literature values. Resulting nominal carbon-based loads were 0.007, 0.09, 1.4, and 7.7 µg LCH g^−1^ OC, and will be used afterwards as treatment names of the LCH AIS experiment. Based on the LCH surface loads, respective water concentrations for the batch experiment were derived, which were 0.17, 1.73, 17.3, and 173 ng L^−1^ during tile exposure to LCH. These water concentrations resulted in equal area loads compared to the AIS experiment. After aufwuchs POC quantification of the batch experiment, loads of 0.009, 0.09, 0.9, and 9 µg LCH g^−1^ OC were calculated, which matched very well with the loads calculated for the AIS experiment and will be used afterwards as treatment names of the batch experiment (Table 1).

**Table 1:** Nominal concentration, area, and aufwuchs loads of LCH in the AIS and the batch experiment

Nominal concentration (ng L^−1^)	Nominal area load (ng cm^−2^)	POC (mg C cm^−2^)	Nominal Aufwuchs load (µg g ^−1^ OC)
*AIS experiment*			
Control	0	0.37 ± 0.04	0
10	2.9	0.37 ± 0.01	7.7
1	0.29	0.21 ± 0.01	1.4
0.1	0.029	0.33 ± 0.02	0.09
0.01	0.0029	0.40 ± 0.03	0.007
*Batch experiment*			
Control	0	0.335	0
171	2.9	0.335	9
17	0.29	0.335	0.9
1.7	0.029	0.335	0.09
0.17	0.0029	0.335	0.009

The aufwuchs loads were calculated based on the POC results of the respective treatments at d_−1_ in the AIS experiment or from the average POC of all the treatments at d_− 1_ in the batch experiment

The chemical analysis of LCH was complex, due to the high lipophilicity of LCH and the resulting fast dissipation from the water as well as the very low concentrations investigated in both experiments. Finally, only the stock solutions were used for the characterization of the exposure during the experiments. The respective subsamples of the LCH stock solutions (0.1 and 1 mL) were transferred into silanized vessels (1.5 mL, Agilent, USA), dried in nitrogen stream (N_2_ 5.0, AIR LIQUIDE), and, afterwards, stored in the refrigerator at 4 °C until analysis. The analysis of LCH was performed with the same method as described for terbutryn. However, different ions were used for the quantification and qualification of two LCH isomers. To quantify LCH ion, 181 *m/z* was used, whereas ions 197 *m/z* and 208 *m/z* were used for qualification. The calculated limit of detection according to DIN 32645 [15] was 150 pg µL^−1^ and the limit of quantification was 500 pg µL^−1^.

### Statistics

Inductive as well as descriptive statistics were performed with the software ‘R’ (ver. 2.11 or higher; [49]). Calculations of concentration–response curves was performed with the software ToxRat-Standard (version 2.10, ToxRat Solutions GmbH, Alsdorf, Germany) using Probit, Logit, or Weibull models, due to the enhanced analytical methods of the software for the calculation of concentration–responses–relationships. In the AIS experiments, the use of formal statistical testing was mostly limited, due to lack of true replication of the AIS [12]. Hence, effect concentrations (No Observed Effect Concentration and Lowest Observed Effect Concentration) were deduced without statistical support. However, the replicated microcosm experiments published in Rybicki et al. [52] can be used to support the results of the terbutryn AIS experiment and the LCH batch experiments for the LCH AIS experiment. The growth of the aufwuchs was evaluated using the area under curve (AUC; [47]) for the development of POC and chlorophyll a. Furthermore, also linear regression was used for aufwuchs and grazers, to calculate net growth rates of the respective organisms in the different AIS treatments.

## Results

### Autumn exposure scenario with the herbicide terbutryn

#### Chemical analysis and water chemistry

The analysis of terbutryn in the AIS experiment at d_0_ revealed concentrations of 0.058 µg L^−1^ (treatment 0.06), 0.51 µg L^−1^ (treatment 0.6), and 5.53 µg L^−1^ (treatment 6), which corresponds in average to 91.2 ± 13.7% of the intended nominal concentration (Table 2). In the lowest treatment (treatment 0.006), the terbutryn concentration was below the limit of detection of the used method. The calculated DT_50_s increased with the terbutryn concentrations and ranged from 63 to 168 days. The control stream was analysed for terbutryn at the end of the experiment and the concentration was below the limit of detection. The analysed physicochemical parameters determined during the experiment showed a similar trend in the control and all terbutryn treatments. No effects of terbutryn on the water chemistry were expected and observed (Table 3).

**Table 2:** Analysed terbutryn concentrations in the water phase at d_0,_ d_27,_ and d_68_ in the different treatments under consideration of the SPE recovery rate of 87.2%

Sampling date	Terbutryn treatments (µg L^−1^)				
	Control	0.006	0.06	0.6	6
d_0_	–	< LOD	0.058	0.51	5.53
d_27_	–	< LOD	0.040	0.39	4.54
d_68_	< LOD	< LOD	< 0.030	0.28	4.19
DT_50_ (day)	n.c.	n.c.	63 (*r*^2^ = 0.98)	77 (*r*^2^ = 0.99)	168 (*r*^2^ = 0.88)
Effective concentration (µg L^−1^)	n.c.	n.c.	0.040	0.38	4.72

The control stream was only analysed for terbutryn at d_68_. DT_50_s were calculated using a non-linear regression model with a first-order dissipation kinetic as reported by Brust et al. [12]. Effective terbutryn concentrations were calculated as geometric mean of all 3 sampling days. *n.c.* parameter not calculated, LOQ = 0.030 µg L^−1^, LOD = 0.007 µg L^−1^

**Table 3:** Median (minimum–maximum) of the physical and chemical parameters determined in the different treatments during the terbutryn experiment

Treatment (µg L^−1^)	Temperature (°C)	Conductivity (µS cm^−1^)	O_2_ (mg L^−1^)	pH	SRP–P (µg L^−1^)	TAN–N (µg L^−1^)	NH_3_–N (µg L^−1^)	NO_3_–N (mg L^−1^)
Control	6.0 (5.8–6.2)	438 (428–452)	11.3 (10.0–11.7)	8.0 (7.9–8.5)	2.2 (< 1–12.1)	7.3 (< 6–19.1)	< 1	3.6 (3.4–3.8)
0.006	5.9 (5.8–6.2)	435 (427–447)	11.1 (9.9–11.4)	8.0 (7.9–8.5)	2.2 (< 1–11.5)	< 6 (< 6–23.7)	< 1	3.5 (3.4–3.8)
0.06	5.8 (5.6–6.0)	440 (429–457)	11.3 (9.9–11.8)	8.0 (7.9–8.5)	2.2 (< 1–12.5)	9.2 (< 6–19.1)	< 1	3.5 (3.4–3.8)
0.6	6.0 (5.8–6.1)	431 (427–443)	11.1 (10.0–11.5)	8.0 (7.9–8.4)	2.0 (< 1–13.4)	< 6 (< 6–31.9)	< 1	3.6 (3.4–3.8)
6	6.0 (5.8–6.2)	438 (428–452)	11.2 (10.1–11.7)	8.0 (7.9–8.5)	2.0 (< 1–11.3)	< 6 (< 6–17.4)	< 1	3.5 (3.4–3.8)

#### Effects of terbutryn on aufwuchs development

**Fig. 2:**
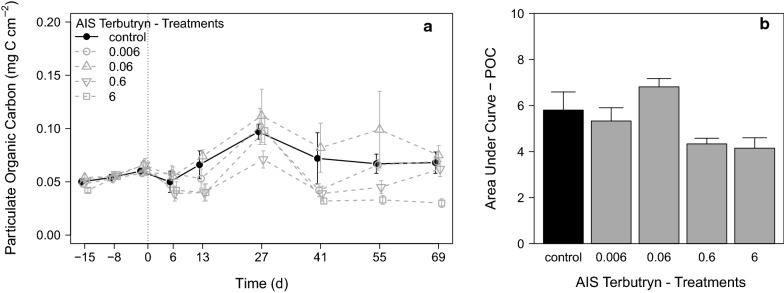
**a** Development of aufwuchs POC during the terbutryn experiment in the different treatments [mean ± SE, *n* = 6]. d_−15_ − d_−1_ pre-application period, d_0_ − d_69_ exposure period. **b** AUC of the POC calculated over the entire experiment [mean ± SE, *n* = 2]. All terbutryn treatments in µg L^−1^

The development of the aufwuchs POC as biomass parameter of the entire aufwuchs community in the AIS during the experiment is shown in Fig. 2a. The lower light availability in November and December led to a slow growth of aufwuchs during the pre-application, and hence, POC increased only slightly from 0.05 ± 0.001 mg C cm^−2^ (d_− 16_) to 0.06 ± 0.002 mg C cm^−2^ (d_− 1_). After addition of grazers at d_− 1_ and application of terbutryn at d_0_, all treatments showed a decrease of POC until d_6_ followed by an increase up to d_27_ without a clear concentration–response–relationship to terbutryn. Afterwards, the control as well as the lowest terbutryn treatments 0.006 and 0.06 showed minor fluctuations of the aufwuchs development and a similar POC amount at the end of the experiment. A clear response to terbutryn in terms of reduced aufwuchs growth was observed in treatments 0.6 and 6. Treatment 6 showed the strongest response to terbutryn, as the POC showed no increase after d_41_ and remained low until the end of the experiment. Similar results were found by the calculated Area Under Curve (AUC) for the POC, which revealed no clear effects in treatments 0.006 and 0.06, but clearly reduced AUCs in the two highest terbutryn treatments 0.6 and 6 (Fig. 2b).

**Fig. 3:**
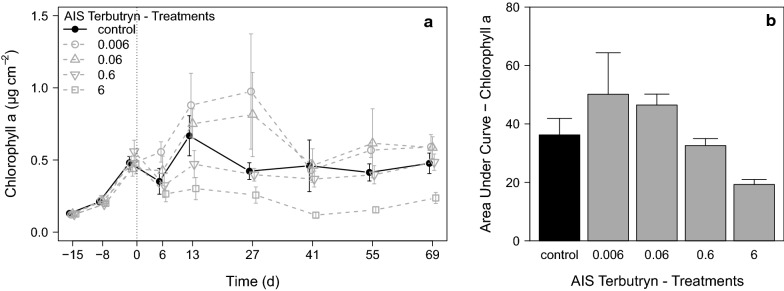
**a** Development of aufwuchs chlorophyll a content during the the terbutryn experiment in the different treatments [mean ± SE, *n* = 6]. d_−15_ − d_−1_ pre-application period, d_0_ − d_69_ exposure period. **b** AUC of the chlorophyll a calculated over the entire experiment [mean ± SE, *n* = 2]. All terbutryn treatments in µg L^−1^

As indicator of the autotrophic fraction of aufwuchs, the chlorophyll a content was determined (Fig. 3a). During the pre-application period, all treatments showed a distinct increase of chlorophyll a from 0.13 ± 0.002 µg cm^−2^ to 0.49 ± 0.02 µg cm^−2^. During the exposure period, the chlorophyll a developed in tendency similar to the POC in all treatments, with a decrease of chlorophyll a until d_6_, a subsequent increase until d_13_ or d_27_ and finally a stable biomass development at the end of the exposure period. A clear concentration–response to terbutryn was only found in treatment 6, which showed a very low chlorophyll a content during the entire exposure period and, finally, only reached 0.27 ± 0.04 µg cm^−2^ at the end of the experiment compared to the control with 0.48 ± 0.07 µg cm^−2^. The calculated AUCs showed a concentration–response relationship to terbutryn, but only treatment 6 showed a distinctly decreased AUC compared to the control (Fig. 3b).

#### Effects of terbutryn on grazer development

**Fig. 4:**
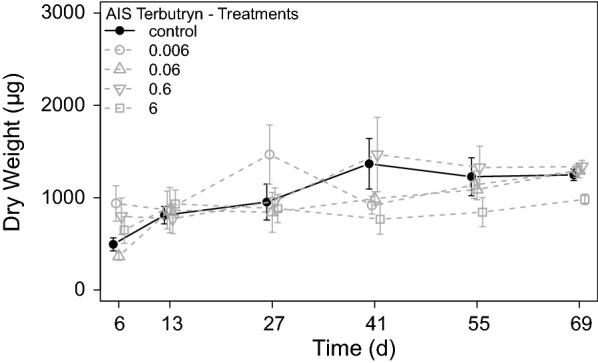
Development of grazer dry weight during the exposure period of the terbutryn experiment in the different treatments [mean ± SE, *n* = 6]. All terbutryn treatments in µg L^−1^

**Fig. 5:**
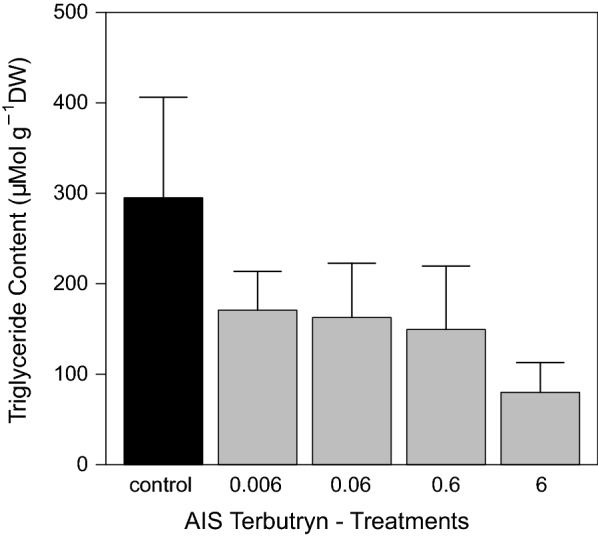
Grazer triglyceride contents at d_69_ in the AIS terbutryn experiment [mean ± SE, *n* = 6]. All terbutryn treatments in µg L^−1^

Grazer mortality stayed below 10% in the control and all terbutryn treatments at the end of the AIS experiment. Hence, no direct acute toxicity of terbutryn occurred during the experiment (data not shown). Nevertheless, effects of terbutryn on grazers were found. Grazer dry weights increased in all treatments during the experiment. While the calculated net growth rate was 8.0 ± 1.3 µg d^−1^ in the control, net growth rates were reduced to only 3.7 ± 1.3 µg d^−1^ in treatment 6. All other treatments showed similar growth rates compared with the control (Fig. 4). The determined triglyceride contents showed a concentration–response relationship with the terbutryn concentration. Control grazers developed the highest triglyceride content in average 295 ± 111 µMol g^−1^ DW, whereas grazers in treatment 6 developed the lowest content of only 80 ± 33 µMol g^−1^ DW. Moreover, grazers of the lower terbutryn treatments developed considerably reduced triglyceride contents compared to the control (Fig. 5).

**Fig. 6:**
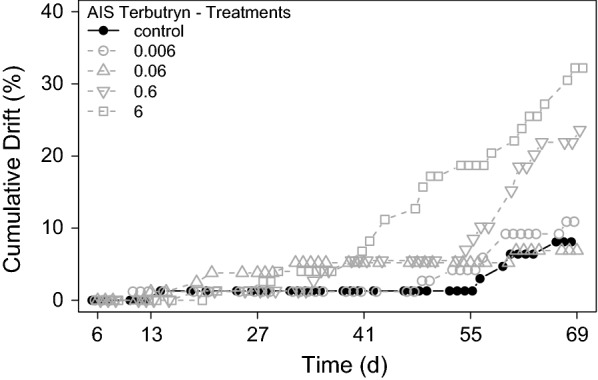
Cumulated overnight drift activity of grazers during the terbutryn experiment. Cumulation started at t_6_ after stabilization of grazer drift caused by adaptation of grazers to the AIS

Finally, the overnight drift of grazers was monitored and analysed by cumulating the drift over the exposure period. During the first week, after introduction of grazers into the AIS (d_− 1_ to d_6_) grazers of all treatments showed a slightly increased and varying drift, but no concentration–response to terbutryn. Considering the varying drift of the first week by excluding all drift before d_6_ from cumulation leads to better comparable drift data (Fig. 6). The results show a low stable drift from d_6_ to d_27_ in all treatments. Afterwards, drift activity increased in all treatments, but strongest responses to terbutryn were found in treatment 0.6 and 6.

### Spring exposure scenario with the insecticide lambda-cyhalothrin

#### Chemical analysis and water chemistry in the AIS and batch experiment

The LCH concentrations in the stock solutions for the AIS and batch experiment roughly matched the range of the intended nominal concentration (Table 4). To consider the deviations of intended and analysed concentrations, we used the nominal concentrations and loads according to Table 1 as conservative approach to describe the exposure in the experiments. Analysed physicochemical parameters determined during the experiment showed no deviations among the control and the LCH treatments and no concentration–response of the water chemistry with the LCH concentration was found (Table 5).

**Table 4:** Nominal and analysed LCH concentrations in the applied stock solutions for the AIS and the batch experiment

LCH treatments (µg g^−1^ OC)	Nominal concentration (µg L^−1^)	Determined concentration (µg L^−1^)	Deviation
*AIS experiment*			
7.7	1	2.29	+ 129%
1.4	0.1	0.097	− 3%
0.09	0.01	0.004	− 60%
0.007	0.001	0.001	± 0%
*Batch experiment*			
9	1	1.172	+ 17.2%
0.9,0.09,0.009	0.1	0.178	+ 78%

In the batch experiment, the three lower treatments were exposed to different volumes of one stock solution

**Table 5:** Median (minimum–maximum) of the physical and chemical characteristics in the different LCH treatments during the AIS and batch experiment

Treatment (µg g^−1^ OC)	Temperature (°C)	Conductivity (µS cm^−1^)	O_2_ (mg L^−1^)	pH	SRP–P (µg L^−1^)	TAN–N (µg L^−1^)	NH_3_–N (µg L^−1^)	NO_3_–N (mg L^−1^)
*AIS experiment*								
Control	6.9 (6–10)	428 (424–430)	11.2 (10.5–14.0)	8.0 (7.9–8.1)	1.8 (1.4–7.7)	13.6 (< 6–80.1)	< 1	3.5 (3.4–3.5)
0.007	6.8 (5.7–9.9)	433 (429–436)	11.4 (10.5–13.9)	8.0 (7.9–8.1)	2.0 (1.4–9.3)	11.5 (< 6–101.1)	< 1	3.5 (3.4–3.6)
0.09	6.8 (5.7–10.1)	430 (423–432)	11.2 (10.3–13.7)	8.0 (7.9–8.1)	2.0 (1.6–8.7)	12.1 (< 6–99.1)	< 1	3.7 (3.6–3.8)
1.4	6.9 (5.9–10.1)	431 (427–434)	11.3 (10.5–13.9)	8.0 (7.9–8.1)	2.2 (1.4–8.9)	13.8 (< 6–52.9)	< 1	3.5 (3.4–3.5)
7.7	6.9 (5.9–10.1)	429 (427–432)	11.4 (10.4–13.9)	8.0 (7.9–8.1)	1.6 (1.4–8.5)	6.8 (< 6–79.2)	< 1	3.5 (3.3–3.6)
*Batch experiment*								
Control	12.7 (11.3–14.6)	658 (616–674)	9.8 (9.4–10.2)	8.1 (8.1–8.2)	n.d.	n.d.	n.d.	n.d.
0.009	12.6 (11.7–14.5)	663 (633–701)	9.9 (9.5–10.3)	8.1 (8.1–8.2)	n.d.	n.d.	n.d.	n.d.
0.09	12.6 (11.3–14.7)	665 (630–701)	9.9 (9.5–10.5)	8.1 (8.1–8.2)	n.d.	n.d.	n.d.	n.d.
0.9	12.5 (11.4–14.8)	662 (625–697)	9.9 (9.5–10.5)	8.1 (8.1–8.2)	n.d.	n.d.	n.d.	n.d.
9	12.6 (11.1–14.6)	650 (631–695)	10.0 (9.5–10.6)	8.1 (8.0–8.2)	n.d.	n.d.	n.d.	n.d.

*n.d.* not determined

#### Effects of LCH on aufwuchs development in the AIS experiment

**Fig. 7:**
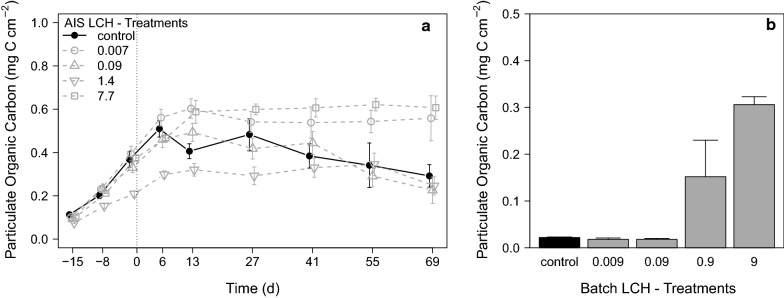
Aufwuchs biomass during **a** LCH AIS experiment [mean ± SE, *n* = 6] and **b** at the end of the LCH batch experiment [mean ± SE, *n* = 4]

Aufwuchs biomasses were determined as POC to analyse indirect effects of LCH. In general, the aufwuchs developed in the LCH AIS experiment much better compared to the AIS terbutryn experiment (Fig. 7a). In the control stream, the aufwuchs biomasses increased from 0.11 ± 0.01 mg C cm^−2^ at d_− 15_ up to 0.37 ± 0.04 mg C cm^−2^ at d_− 1_. All other streams developed equally except of one stream, which achieved only a biomass of 0.21 ± 0.01 mg C cm^−2^ at d_− 1_. After exposure to LCH, all treatments showed a further increase of aufwuchs biomass until d_6_. Afterwards, the control as well as treatment 0.09 showed a decrease of POC until the end of the experiment. All other treatments stayed at their respective aufwuchs level with only slight variations. A clear concentration–response relationship to LCH loads was not observed, although treatment 7.7 µg g^−1^ OC showed the highest aufwuchs biomass. However, the high biomass at 0.007 µg g^−1^ OC and the delayed aufwuchs development in treatment 1.4 µg g^−1^ OC masked a clear concentration–response relationship.

#### Effects of LCH on grazer development in the AIS experiment

**Fig. 8:**
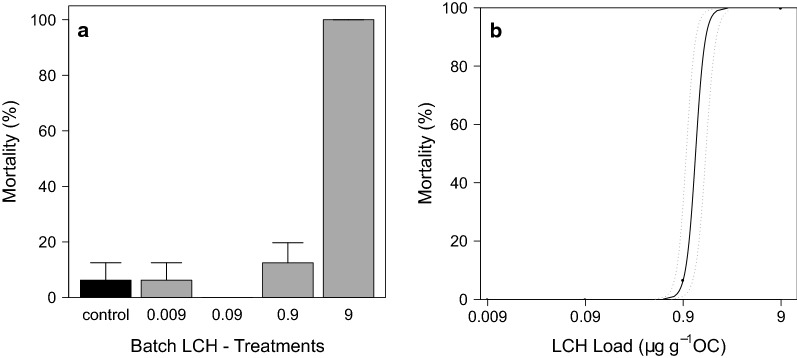
**a** Grazer mortality at the end of the LCH AIS [mean, *n* = 1] and **b** Concentration response relationship calculated using Probit model after correction of control mortality according to Abbott. Dotted lines indicate the 95 % confidence level. Treatment names according to the nominal LCH loads in µg g^−1^ OC as described in Table 1

Grazer mortality showed a clear-and-steep concentration–response relationship with the LCH load/concentration, whereas mortality remained low in the control (7.7%) and up to 0.09 µg g^−1^ OC, 100% mortality was observed at the highest LCH load of 7.7 µg g^−1^ OC (Fig. 8a). LC_*x*_ values were derived using a probit model after correction of control mortality according to Abbott. LC_50_ and LC_10_ (69 days) were 2.42 µg g^−1^ OC (CI 95 0.54–11.59) and 0.86 µg g^−1^ OC (CI 95 0.01–2.13), respectively (Fig. 8b). As exposure occurred over the water phase during the AIS experiment, also the water concentrations can be used. Calculated effect values for the water concentrations using a two-parameter logit model and after correction of control mortality were 1.23 ng L^−1^ (CI 95 1.15–1.13) and 0.97 ng L^−1^ (CI 95 0.91–1.05), for the LC_50_ and LC_10_ (69 days), respectively (data not shown).

**Fig. 9:**
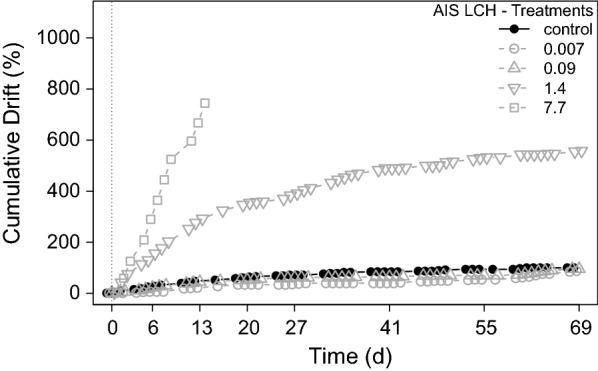
Cumulated overnight drift activity of grazers during the LCH experiment. All LCH treatments in µg g^−1^ OC

Distinct LCH effects were observed in the behavioural parameter overnight drift. To account for increased drift of grazers due to adaptation to the streams during the first week, as observed in the terbutryn experiment, grazers were introduced earlier in the pre-application period at d_− 8_. Grazers in all streams showed a similar low-drift activity at the time of exposure to LCH, and hence, cumulation was started with the time of exposure (d_0_). Approximately 3 h after addition of LCH to the streams, a clear increase of drift was observed in the highest LCH treatment (7.7 µg g^−1^ OC) and a slight increase in the second highest (1.4 µg g^−1^ OC) treatment. Drift remained low in all other treatments. The cumulated overnight drift activity showed a clear concentration–response to LCH, respectively (Fig. 9).

**Fig. 10:**
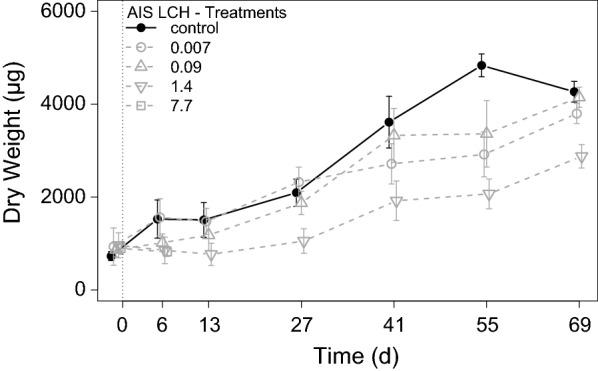
Development of grazer dry weights during the LCH AIS experiment in the different treatments [mean ± SE, *n* ≥ 6]. All LCH treatments in µg g^−1^ OC

**Fig. 11:**
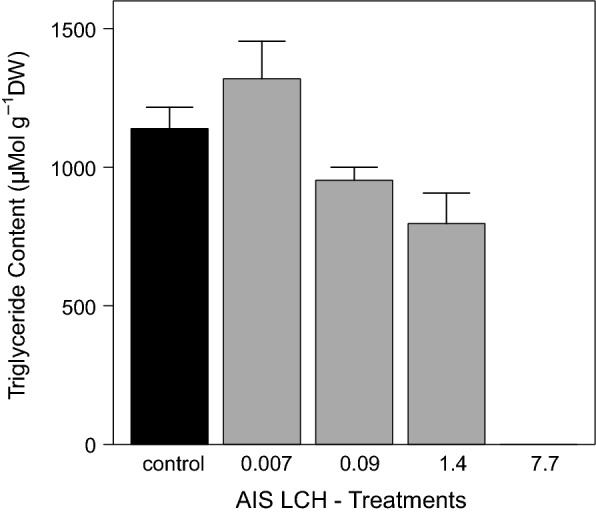
Grazer triglyceride contents at the end of the LCH AIS experiment in the different treatments [mean ± SE, *n* = 6]. No triglyceride levels were determined in the highest LCH treatment, due to total mortality. All LCH treatments in µg g^−1^ OC

Calculated net growth rates of grazers over the exposure period were clearly reduced at 1.4 µg g^−1^ OC with 27.6 ± 3.3 µg d^−1^ compared to the control with 52.5 ± 3.3 µg d^−1^ and the other low LCH treatments (Fig. 10). Growth of grazers within the highest exposure treatment was negligible with only 4.0 ± 50.7 µg d^−1^ and limited to the first week of the exposure period, as all grazers of this treatment were removed, due to mortality latest at d_7_. Finally, the analysed triglyceride contents of grazers determined at the end of the experiment showed a concentration–response. Whereas the control and treatment 0.007 µg g^−1^ OC developed triglyceride contents of 1139 ± 77 µMol g^−1^ and 1319 ± 136 µMol g^−1^, respectively. Triglyceride levels in treatments 0.09 µg g^−1^ OC and 1.4 µg g^−1^ OC achieved only 953 ± 47 µMol g^−1^ and 796 ± 110 µMol g^−1^ (Fig. 11).

#### Effects of LCH on aufwuchs development in the batch experiment

In contrast to the AIS experiment, a clear concentration–response relationship of aufwuchs with LCH loads was observed in the batch experiment, whereas very low aufwuchs biomasses were determined in the control as well as in treatments 0.009 and 0.09 µg g^−1^ OC. An increase of the aufwuchs POC was observed in treatment 0.9 and 9 µg g^−1^ OC (Fig. 7b). The statistical evaluation of the data revealed a clear general effect of LCH (Kruskal–Wallis–ANOVA, *df* = 4, χ^2^ = 11.70, *p* = 0.022). However, no significant deviations of the single LCH treatments from the control in the performed Post hoc test (Nemenyi test for multiple non-parametric comparisons) were found.

#### Effects of LCH on grazer development in the batch experiment

**Fig. 12:**
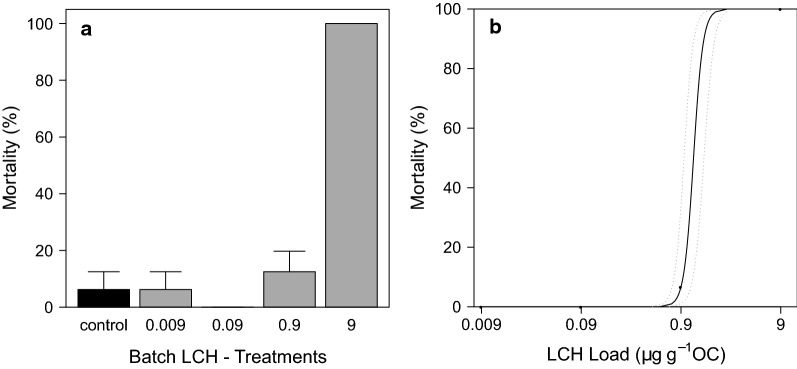
**a** Grazer mortality at the end of the batch experiment [mean ± SE, *n* = 4] and **b** Concentration response relationship calculated using 2-parameter Logit model after correction of control mortality according to Abbott. Dotted lines indicate the 95 % confidence level. Treatment names according to the nominal LCH loads in µg g^−1^ OC as described in Table 1

**Fig. 13:**
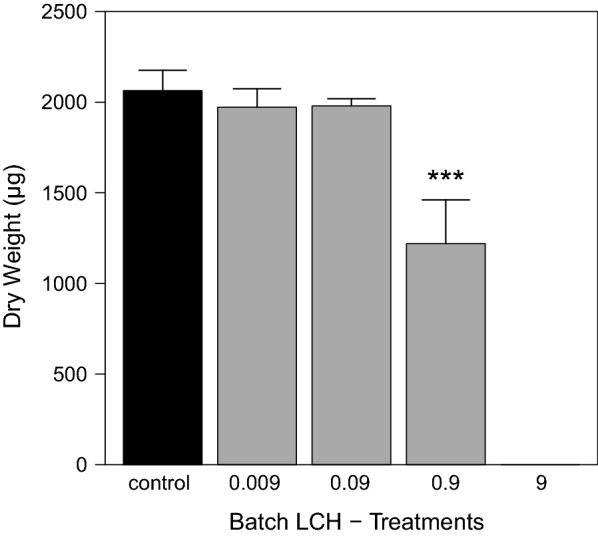
Grazer dry weights at the end of the LCH batch experiment [mean ± SE, *n* = 4]. All LCH treatments in µg g^−1^ OC. *** significantly different from control with *p* < 0.001

**Fig. 14:**
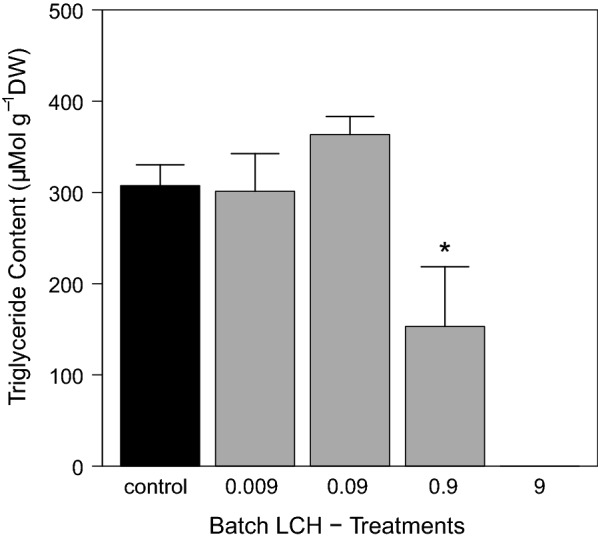
Grazer triglyceride contents at the end of the LCH batch experiment [mean ± SE, *n*=4]. All LCH treatments in µg g^−1^ OC. * significantly different from control with *p* < 0.01.

In the batch experiment, a clear concentration–response relationship of LCH to grazer mortality was found. In the control, mortality remained low at only 6.25 ± 6.25%, whereas total mortality was observed in the treatment with the highest LCH load of 9 µg g^−1^ OC (Fig. 12a). The calculated LC_50_ and LC_10_ values (28 d) were 1.20 µg g^−1^ OC (CI 95 0.96–1.53) and 0.95 µg g^−1^ OC (CI 95 0.77–1.19), respectively (Fig. 12b). Effect values for the water concentration were not calculated, as exposure was performed over exposed tiles and not directly over the water phase as described in the methods section. Grazer dry weights and triglyceride contents showed both a clear concentration–response relationship with the LCH loads (Figs. 13, 14). A significant reduction compared to the control was found for both endpoints at 0.9 µg g^−1^ OC (dry weight: ANOVA with Box–Cox transformed data, *df* = 3, *F* = 7.75, *p* = 0.004—contrast analysis: control vs. 0.9, *t* value = − 4.31, *p* = 0.001; triglyceride content: ANOVA, *df* = 3, *F* = 4.69, *p* = 0.02—contrast analysis: control vs. 0.9, *t* value = − 2.63, *p* = 0.02).

## Discussion

In the present study, batch and artificial indoor stream (AIS) experiments using a tiered approach were performed. The aim was to investigate direct and indirect effects of herbicide and insecticide exposure on the development of the benthic grazer *R.* *semicolorata.* The exposure scenarios were designed to represent realistic scenarios occurring in the natural aquatic environment at the respective life span. Clear indirect effects on grazers were found on drift, growth, and triglyceride levels in the winter exposure scenario with the herbicide terbutryn at 0.38 µg L^−1^, as a consequence of reduced aufwuchs quantity. Clear-and-steep concentration–response relationships of lambda-cyhalothrin (LCH) exposure with lethal and sub lethal effects on grazers were obvious. Clear direct effects on growth, triglyceride levels, and drift were observed at concentrations close to the LC_50_ of 2.42 µg g^−1^ OC. An indirect effect on the aufwuchs was found in the batch experiment as a result of reduced feeding activity of grazers caused by LCH exposure.

### Chemical analysis and physicochemical characteristics in the experiments

In the AIS experiment, the analysis of terbutryn in the control revealed a concentration below the limit of detection. The nominal concentrations of terbutryn in treatments 0.06, 0.6, and 6 µg L^−1^ have been achieved well 1 h after application. In treatment 0.006, only traces of terbutryn were quantified, due to the low intended concentration, which was already at the beginning of the experiment below the calculated limit of detection of the analytical method. Terbutryn showed slow and concentration dependent dissipation from the water phase with DT_50_ values between 63 and 168 days^−1^. Dissipation was expected, due to the results of the previous experiments [12, 41, 52]. However, the reduced aufwuchs biomass in the AIS experiment decreased the dissipation by a factor of 2–6 from expected 28 day^−1^ to the observed times. Hence, calculated effective concentrations were 85% of the initial concentration in treatment 6 or at least 68% in treatment 0.06. A stable exposure of all organisms during the entire terbutryn experiment was herewith realised. An impact of terbutryn on the water chemistry as observed at high concentrations by Brust et al. [12] was not evident. The determined physicochemical characteristics of the water were in a range of former micro- and mesocosm experiments [12, 36, 52] and suitable for aufwuchs and grazers.

To investigate the effects of insecticides on grazers in spring, a second scenario was investigated using LCH. Due to the high log K_OW_ of 7, a fast dissipation from the water phase to biofilms was expected and had been observed in former studies [6, 17, 62]. The calculation of the exposure and subsequent analysis was, therefore, more complex, while the concentration analysis in the biofilm was not undertaken, due to the very low investigated concentrations and the limits of detection of the analytical method. Consequently, the low concentrations of LCH used led to a reduced reliability of the chemical analysis, as revealed by the high variation of the analysed stock solutions. However, the analysis generally verified the respective concentrations of LCH in the stock solutions applied. The water exposure to LCH was limited to a short period, as LCH rapidly adsorbed to the aufwuchs, which was apart from some grazer faeces, the major source of organic carbon available in the streams. LCH degradation is reported [19, 24], and hence, considering the ambient conditions in the experiments, minor degradation of LCH can take place. As LCH exposure was difficult to describe, we chose a conservative approach using the nominal concentrations and the, respectively, derived aufwuchs loads for the streams and the batch experiment, as explained above (Table 1). Determined physicochemical characteristics of the water were similar to the terbutryn experiment and, hence, suitable for the test organisms.

### Effects of herbicide exposure on early juvenile grazers in autumn and winter

The AIS terbutryn experiment investigated the effects of late-autumn herbicide exposure on early life stages of the grazer *R.* *semicolorata*. As expected for an herbicide and observed in the previous experiments, terbutryn directly affected the aufwuchs community [12, 52]. Overall, aufwuchs biomasses remained low compared to the previous experiments [12, 36, 52], probably due to the low-light availability in autumn and reduced water temperatures used in the present study. Nevertheless, a clear concentration–response of terbutryn with the aufwuchs biomass in terms of POC and Chlorophyll a was observed during the exposure period. Using the AUC, as suggested by Brust et al. [12], revealed apparent effects of terbutryn starting at 4.72 µg L^−1^ for Chlorophyll a and 0.38 µg L^−1^ for the aufwuchs POC content, which is in line with the former terbutryn studies [52]—LOEC 6 µg L^−1^; [12]—LOEC 0.43 µg L^−1^; [21, 23]—LOEC < 10 µg L^−1^).

Effects on aufwuchs quality, due to a shift of the aufwuchs composition, could be expected as former experiments revealed clear effects of terbutryn on aufwuchs composition in the tested concentration range [21, 23]. In addition, own previous studies [52] using similar aufwuchs clearly revealed an increase of cyanobacteria biomass in the community. Considering that cyanobacteria reduce the nutritional value of the aufwuchs for grazers, due to production of toxins, digestion-resistant cell walls, low unsaturated fatty acid contents, and their often filamentous growth form [1, 22, 40], further negative effects on grazers development seem possible.

As discussed in Rybicki et al. [52], no acute toxicity of terbutryn in the investigated concentration range on the grazer *R.* *semicolorata* was expected, which was confirmed by low mortality of < 10% in all the treatments. Nevertheless, sub lethal effects of terbutryn on grazers cannot be excluded. Different studies reported sub lethal effects of terbutryn and other triazine herbicides, e.g., on physiological or behavioural endpoints in vertebrates and invertebrates at very low concentrations [57, 63]. However, reported sub lethal effects especially on invertebrates always coincided with increased mortality in those studies, which was not found in the present study. Nevertheless, clear concentration–response relationships of different grazer endpoints with the terbutryn concentration were found in the present experiment probably caused by indirect effects rather than direct toxicity.

A clear response to terbutryn was observed for the behavioural endpoint overnight drift. A usually induced catastrophic drift, as often observed after pesticide exposure in the environment [10], was not observed shortly after terbutryn exposure. In contrast, drift increased during the last weeks of the experiment in the two highest terbutryn treatments (Table 6). Abiotic factors influencing drift like flow velocity or substratum [10, 45] can be excluded for the present experiment, as they were equal in all AIS. However, biotic factors like competition or available amount of food are more likely to cause the observed increase of drift [10]. Different studies observed a clear correlation of food availability, subsequent competition, and drift of macroinvertebrates [8, 27, 34]. Considering the determined aufwuchs biomasses in the different treatments reveals an obvious food depletion with increasing terbutryn concentration in the present experiment, which matches with the observed late increase of grazer drift. The probable shift of the aufwuchs community towards cyanobacteria, as discussed above, may even increase the drift in the terbutryn treatments. Hence, an indirect effect of terbutryn via food quantity and quality seems most probable to explain the concentration–response observed for grazer drift.

**Table 6:** Deduced effect concentrations of terbutryn for the AIS experiment of the different test organisms and observed endpoints

Organism	Endpoint	NOEC	LOEC
*AIS experiment*			
Grazer	Overnight drift	0.04 µg L^−1^	0.38 µg L^−1^
	Dry weight	0.38 µg L^−1^	4.72 µg L^−1^
	Triglyceride level	0.38 µg L^−1^	4.72 µg L^−1^
Aufwuchs	POC	0.04 µg L^−1^	0.38 µg L^−1^
	Chlorophyll a	0.38 µg L^−1^	4.72 µg L^−1^

A concentration–response was also found for the important endpoint grazer growth, which was monitored over the entire experiment. The calculated growth rate of 8 µg day^−1^ in the control was considerably lower compared to the previous experiments where growth rates of about 70 µg day^−1^ were found [52]. These generally reduced growth rates in all treatments can be attributed to the generally low aufwuchs biomass during this experiment, which may induce a slight starvation in all treatments independent from terbutryn. Furthermore, the more complex ambient conditions of the present experiment, e.g., the flow velocity, cause a higher energy expenditure for grazers [45], while metabolic processes of poikilothermic grazers are reduced, due to the lower temperature in the AIS compared to former experiments. However, a comparison between the single treatments revealed a clearly reduced growth rate in treatment 6 compared to the control (Table 6), which is an indication of even stronger starvation in this treatment and matches well with the results of the aufwuchs biomass.

Finally, the triglyceride contents as marker of the physiological condition of grazers [33, 68] were determined at the end of the exposure. All larvae in the terbutryn treatments developed a lower triglyceride content compared to the control. However, for the interpretation, the large standard error of the control must be considered. Only treatment 6, which developed only one-third of the value compared to the control, showed a distinct reduction and thus strong indication of starvation induced by terbutryn. This matches well with the reduced growth of grazers as well as the reduced aufwuchs biomass in this treatment (Table 6). A reduction of triglyceride levels as indirect effect of terbutryn has been observed in the previous experiments, too. Rybicki et al. [52] have determined an LOEC_TG_ of 0.21 µg L^−1^, which is in a similar range as in the present experiment, showing that triglyceride contents are very sensitive endpoints of grazers.

Using the herbicide terbutryn as representative of this pesticide class, our first hypothesis was that environmentally relevant concentrations of terbutryn are able to affect the development of the early life stages of *R. semicolorata* negatively, due to food shortage and reduced food quality. Table 6 summarizes the deduced effect concentrations from this experiment, which clearly show the indirect effects of terbutryn on grazers as initially hypothesized. These data, together with environmental concentrations, have to be implemented to a risk assessment to estimate the impact on grazers in the environment.

Although terbutryn is currently not approved in agricultural (European Council Directive 91/414/EEC, European Commission Regulation 2076/2002), it is still regularly detected in German surface waters, due to the use as biocide in antifouling paints or as protection agent for fibres and textiles (EU directive 98/8/EG). The environmental quality standard of 0.34 µg L^−1^ (2013/39/EU) is thereby punctually exceeded and maximum concentrations reported achieve up to 5.6 µg L^−1^ in Hesse [48] and more recently up to 1.9 µg L^−1^ in the Free State of Saxony (2011, Surface water monitoring of the Federal Agency for Environment, Agriculture and Geology, Saxony). From a regulatory point of view, using 1.9 µg L^−1^ as Measured Environmental Concentration and the lowest NOEC_Drift_ of 0.04 µg L^−1^ for the calculation of the Toxicity Exposure Ration (TER) results in a quotient of 0.02. This indicates a clear risk of terbutryn for the grazer *R.* *semicolorata* in the environment and supports our first hypothesis. Considering the broad usage of herbicides in agriculture (33% of spread pesticides in Europe; Eurostats, Pesticide sales Reg. 1185/2009), e.g., glyphosate, metazachlor, and others, indicates a general risk and threat of this substance class for the aquatic ecosystems and specifically for grazers, which is in line with the observations of Schaefer et al. [53] for large rivers in north Germany.

### Effects of insecticide exposure on semi-juvenile grazers in the early spring

The second set of experiments, encompassing the AIS and the batch experiment, aimed on possible direct effects of insecticide exposure on the grazer *R.* *semicolorata* during spring. Due to the known fast dissipation of LCH from the water phase into the aufwuchs, an exposure route via food or contact of grazers to the aufwuchs was expected [35]. Therefore, LCH load per tile area was calculated to have a comparable exposure within both experiments and account for varying volume-to-surface ratios. The determined LC_50_ and LC_10_ values of 2.42 µg g^−1^ OC and 0.86 µg g^−1^ OC in the AIS are in accordance with the values derived from the batch experiment with 1.2 µg g^−1^ OC and 0.95 µg g^−1^ OC, respectively. This similarity indicates that the used approach of calculating surface and aufwuchs loads was successful, although surface–volume ratio differed significantly between both experimental setups. Suitable conditions for grazers in both experiments are indicated by control mortalities < 10%. Determined effect values matches well with LC_50_ (10 d) values of 2.8 µg g^−1^ OC reported by Maul et al. [38] for sediment exposure of *Chironomus* *riparius*. Chronic effect values for sediment exposure of mayflies are scarce, but values based on the initial water concentrations are available from Wiberg-Larsen et al. [67] and Schroer et al. [55]. Wiberg-Larsen et al. [67] exposed different mayflies and other macroinvertebrates for 90 min to LCH and determined afterwards the mortality over a period of 7 days. The resulting LC_50_s (6 days) were in a range of 140 (*Caenis* *horaris*) to 9350 ng L^−1^ (*Ephemera* *danica*), which is close to the reported acute effect values of LCH reported by Schroer et al. [55]. However, Schroer et al. [55] also performed more complex mesocosm experiments and reported EC_50_ values (10 d) for the endpoint population development of 24.0 ng L^−1^ and 14.3 ng L^−1^ for the mayflies *Cloeon* *dipterum* and *Caenis* *horaris*, respectively. These values are much closer to the LC_50_ determined in the current AIS experiment using the initial water concentration, which was 1.23 ng L^−1^ (nominal). The obvious variability of the effect values even within the same species indicates that the use of water concentrations for comparison can be misleading, due to the difficult physicochemical properties of LCH and its fate and behaviour in the experiment. This supports our decision to calculate surface and aufwuchs loads. A recent study of Pristed et al. [46] investigated the differences between water and biofilm exposure of LCH to the mayfly *Heptagenia* *sulphurea* and found no strong effects during exposure via aufwuchs, but clear effects after exposure via the water phase. This is in contrast to our findings of the LCH batch experiment, where aufwuchs covered tiles were exposed for 24 h to LCH and then transferred to the final exposure vessels. An exposure via water phase was excluded by this methodology; hence, observed mortality was clearly caused by exposure via food or by direct contact of grazers to the contaminated aufwuchs. The obvious difference in the outcome compared to Pristed et al. [46] may be caused by the different exposure times of aufwuchs to LCH. Considering that dissipation from water is mainly the result of sorption to organic fractions in the system and that sorption mainly results from the law of mass action reveals that the longer exposure time in the present batch experiment (24 h) is more suitable to bind substantial fractions of LCH to aufwuchs compared to Pristed et al. [46].

Apart from grazer mortality, further sub lethal effects on grazers were observed. It was shown that the overnight drift determined during the AIS LCH experiment was a sensitive endpoint, as it revealed a clear sub lethal effect of LCH at 1.4 µg g^−1^ OC, which is in the range of the calculated LC_50_ of grazers. It is noticeable that drift was thereby a very fast-responding behavioural endpoint and markedly increased already 3 h after exposure in the two highest treatments (7.7 and 1.4 µg g^−1^ OC). This is consistent with the observations of Norum et al. [43] and Heckmann and Friberg [25], who observed catastrophic drift of mayflies after LCH exposure, too. Drift can be an active process, e.g., to avoid predation, starvation, intoxication, or even competition [8, 10, 69] or a passive process due to behavioural dysfunction or paralysis. The experimental design of the AIS experiment did not allow distinguishing active and passive drift. However, we estimate that both processes occurred during the experiment. While grazers at 7.7 µg g^−1^ OC showed obvious signs of paralysis shortly after exposure, the drift increased only slowly in treatment 1.4 µg g^−1^ OC without signs of paralysis and decreased over the further course of the experiment, which indicate an active drift process. These results underline the importance to include sensitive fast-responding behavioural endpoints like the drift into the ecotoxicological risk assessment [67], which are able to indicate negative impacts on the ecosystem even in the sub lethal concentration range.

The determined developmental parameters were in line with the above-discussed observations. In both experiments, grazers showed clear concentration–response relationships of LCH with growth of the grazers at concentrations around 1 µg g^−1^ OC (Table 7). A similar concentration–response relationship with LCH was found for the triglyceride content in the AIS and batch experiment. Whereas, in the batch experiment, a significant reduction of triglyceride levels at 0.9 µg g^−1^ OC was determined, the results in the AIS experiment showed a slight reduction already at 0.09 µg g^−1^ OC. This is a result of the longer exposure time of 69 day^−1^ compared to 28 day^−1^ in the batch experiment. Considering the low mortality in the treatments with reduced triglycerides levels indicates either a sub lethal effect of LCH on the feeding behaviour of grazers, as observed for different aquatic invertebrates by Palmquist et al. [44] in experiments with the pyrethroid esfenvalerate, or even an altered energy allocation pattern, due to increased costs of detoxification [38, 56].

**Table 7:** Deduced effect concentrations of LCH during the AIS and batch experiments of the different test organisms and observed endpoints

Organism	Endpoint	NOEC	LOEC	LC_10/50_
*AIS experiment*				
Grazer	Mortality	–	–	0.86/2.42 µg g^−1^ OC 0.97/1.23 ng L^−1^
	Overnight drift	0.09 µg g^−1^ OC	1.4 µg g^−1^ OC	–
	Dry weight	0.09 µg g^−1^ OC	1.4 µg g^−1^ OC	–
	Triglyceride level	0.007 µg g^−1^ *OC*	0.09 µg g^−1^ OC	–
Aufwuchs	POC	n.d.	n.d.	–
*Batch experiment*				
Grazer	Mortality	–	–	0.95/1.2 µg g^−1^ OC
	Dry weight	0.09 µg g^−1^ *OC*	0.9 µg g^−1^ OC	–
	Triglyceride level	0.09 µg g^−1^ *OC*	0.9 µg g^−1^ OC	–
Aufwuchs	POC	0.09 µg g^−1^ *OC*	0.9 µg g^−1^ OC	–

*n.d.* not deduced, due to missing concentration–response relationship

The aufwuchs POC data of the LCH batch experiment support the feeding inhibition hypothesis. The results show a clear increase of aufwuchs biomass in the 0.9 µg g^−1^ OC treatment compared to the lower LCH treatments and the control. As the mortality remained low within this treatment, an effect due to mortality (density mediated), as in the highest LCH treatment, can be excluded and the cause of the decreased grazing efficiency must be an effect on the feeding behaviour (trait-mediated effect), which matches well with the results of dry weight and triglyceride levels. Unfortunately, the data distribution in the LCH batch experiment prevented a clear statistical deduction of this indirect effect of LCH on aufwuchs. However, due to the obvious differences of the POC between the control and the two highest LCH treatments, an NOEC_Aufwuchs_ at 0.09 µg g^−1^ OC and an LOEC_Aufwuchs_ at 0.9 µg g^−1^ OC were at least estimated (Table 7). This indirect effect of LCH on the aufwuchs biomass was finally not clearly visible in the AIS experiment, due to the unknown initially reduced aufwuchs growth in treatment 1.4 µg g^−1^ OC and the very high aufwuchs growth in treatment 0.007 µg g^−1^ OC, which finally masked any statistical deduction of indirect LCH effects on the aufwuchs. However, the clear POC difference between the control and the highest LCH treatment at least indicate the presence of this indirect effect.

Within this set of experiments, we investigated the hypothesis that environmentally relevant concentrations of LCH cause significant behavioural changes, which results in increased drift and reduced feeding. The experiment clearly revealed the occurrence of lethal and sub lethal effects (Table 7). Within the batch experiment, even a density and trait-mediated indirect effect of LCH on the aufwuchs was quantified.

The determination of LCH in the environment is complex, due to its high log K_OW_ and the resulting fast dissipation by adsorption to organic fractions and surfaces, which is often used as argument for the environmental compatibility of LCH. However, LCH does not disappear from system, but relocate to another environmental compartment, as obvious from the batch experiment, where it is bioavailable for epi- and endobenthic organisms [2, 38, 44, 64]. To our knowledge, no sediment concentrations of LCH in Germany are published. However, the current investigations of Bereswill et al. [7] revealed a high detection frequency and high Toxic Unit values (referred to *Daphnia* *magna* acute toxicity) of LCH in German surface waters and runoff samples. LCH was found in the runoff from agricultural fields in average concentrations of 23 µg kg^−1^ DW, which corresponds to a concentration of 1.53 µg g^−1^ OC using the reported organic fraction of the soil of 1.5%. The detected maximum concentrations reach even 5.8 µg g^−1^ OC (88 µg kg^−1^ DW), hence, exceeding the LC_50_ values determined in the presented experiment. The runoff values match well with sediment concentrations from the USA reported by Weston et al. [64] and Amweg et al. [3], which determined concentrations up to 1.68 µg g^−1^ OC in river sediments. Using the maximum runoff concentrations reported by Bereswill et al. [7] as MEC and the lowest NOEC of 0.007 µg g^−1^ OC reveals a TER value of 0.001 indicating a clear risk of LCH for the grazer *R.* *semicolorata* in the environment, hence, supporting our initial hypothesis, that LCH exposure at environmentally relevant concentrations causes distinct sub lethal effects in grazers. Moreover, even lethal effects seem possible.

### Grazer life-history and implications for the risk assessment

The used grazers provide an important function for stream ecosystems by controlling the aufwuchs biomass at the streambed [4, 18, 22, 29, 31], which is especially important during the aufwuchs biomass peak in spring before foliation of deciduous trees to prevent/reduce external biological colmation [30, 50]. A successful provision of this function, however, requires a suitable biomass of grazers [4] at a specific time in spring. The performed experiments revealed specific effects of pesticides during the grazer life cycle, which are able to influence the survival, spatial distribution (drift), growth, and even fecundity (triglycerides) of grazers and, hence, their functional performance in spring. A long-lasting reduction of grazer growth rates during autumn or winter caused by herbicide-induced starvation as observed in the terbutryn experiment may led to a mismatch of the grazer and aufwuchs biomass peak in spring. Furthermore, sublethal effects of insecticides as observed in the LCH experiments are likely to influence the grazing performance either directly, e.g., via feeding inhibition or indirectly by affecting the physiological condition of grazers and, subsequently, their stress tolerance [56] or even fecundity [52]. Of course, the possibility of compensatory growth and recovery after a phase of increased stress, as observed in other studies with aquatic invertebrates [13, 26], needs to be considered. Environmental growth data for the grazer *R.* *semicolorata* provide strong indications of the existence of a compensatory feeding behaviour after starvation [68]. However, such complex interactions are not included in the standard risk assessment and are even difficult to assess in higher tier approaches. Considering that, in a time-shifted exposure scenario with herbicide and insecticides, the final physiological state of grazers after herbicide exposure is the starting point for the insecticide exposure reveals the possibility of a synergistic effect between both exposures and, hence, an even higher complexity. A synergistic effect of the herbicide exposure mediated by the physiological condition of grazers and resulting reduced stress tolerance may increase the risk of grazers during the subsequent insecticide exposure. However, such time-shifted exposures are currently not considered within the regulation process of pesticides. The results of the present experiments strongly indicate that, such scenarios, under consideration of the life-history of the used test organism, can reveal important information to understand the complex effects of pesticides in the aquatic environment. Especially uni- and semivoltine macroinvertebrates, which complex life cycles hampers their regular use for the standard risk assessment of pesticides, seem to be susceptible to time-shifted exposures and may fail to perform their functional role within the aquatic ecosystem.

## Conclusion

The performed artificial indoor stream experiments clearly revealed direct and indirect negative effects of herbicides and insecticides on the grazer *R.* *semicolorata* at environmentally relevant exposure scenarios as shown by the calculated TER values. In general, the observed effects are very likely to affect the important ecosystem function of benthic grazing. One major aspect mainly neglected in hazard identification is a time-shifted exposure scenario. From our experiments, it is evident that effects at different life stages could be synergistic and, hence, increase the risk. This especially concerns uni- and semivoltine macroinvertebrates, which spend long periods with consecutive pesticide exposure in a respective environment. Therefore, we suggest to perform further research with time-shifted consecutive exposure scenarios to gain a better understanding of the complex interactions of pesticides with the life cycle and the food webs of macroinvertebrates.
